# Ethanolic extract of *Brucea javanica* inhibit proliferation of HCT-116 colon cancer cells *via* caspase activation

**DOI:** 10.1039/c7ra09618f

**Published:** 2018-01-02

**Authors:** E. Bagheri, F. Hajiaghaalipour, S. Nyamathulla, N. A. Salehen

**Affiliations:** Department of Pharmacy, Faculty of Medicine, University of Malaya 50603 Kuala Lumpur Malaysia Elhambagheri@siswa.um.edu.my Nurain_36@um.edu.my; Institute of Biological Science, Faculty of Science, University of Malaya 50603 Kuala Lumpur Malaysia; Department of Biomedical Science, Faculty of Medicine, University of Malaya 50603 Kuala Lumpur Malaysia

## Abstract

*Brucea javanica* (L.) Merr. is a well-known plant in Chinese System of Medicine. Its fruits and seeds have been reported to possess curative properties against various ailments. The chemical constituents and biological activity of this plant have been an interesting area in plant and chemistry medicine. The aim of this study is to evaluate the antiproliferative effects of the *B. javanica* extract against a colon cancer cell line and identification of the chemical components derived from the extract. An ethanolic extract from *B. javanica* fruits was prepared by cold maceration method, subjected to LC-MS profiling to elucidate the composition abbreviated as BJEE. The extract was screened for the cytotoxicity effects on HCT-116 colon cancer cells *via* MTT and LDH methods. Additionally, AO/PI staining verified apoptosis features in HCT-116 cells through microscopic analysis. ROS, caspase activity, and gene expression has been performed to identify its possible mechanism of actions which contribute to apoptosis. Output data from this study showed BJEE inhibited the cell proliferation of HCT-116 colon cancer cells at IC_50_ value of 8.9 ± 1.32 (μg mL^−1^) and significantly increased the levels of caspase-8, 9, and 3/7 in treated cells in comparison to untreated. The changes in expression of caspase genes and some apoptosis genes like Bax and Bcl-2 were confirmed using RT-PCR. Phytochemical analysis by LC-MS identified six major active compounds (bruceine D, isobrucein A, quassimarin, C16 sphinganine, phytosphingosine, and enigmol) in BJEE that may play a key role in cell apoptosis. The current study showed BJEE could be a promising agent for colorectal cancer therapy by significant increase in caspase activity level, and up-regulation of the specific apoptotic genes.

## Abstract 2

*Brucea javanica* ethanolic extracts induced apoptosis in colon cancer cell line (HCT-116) *via* mitochonderial and extrinsic pathways.

## Graphical abstract

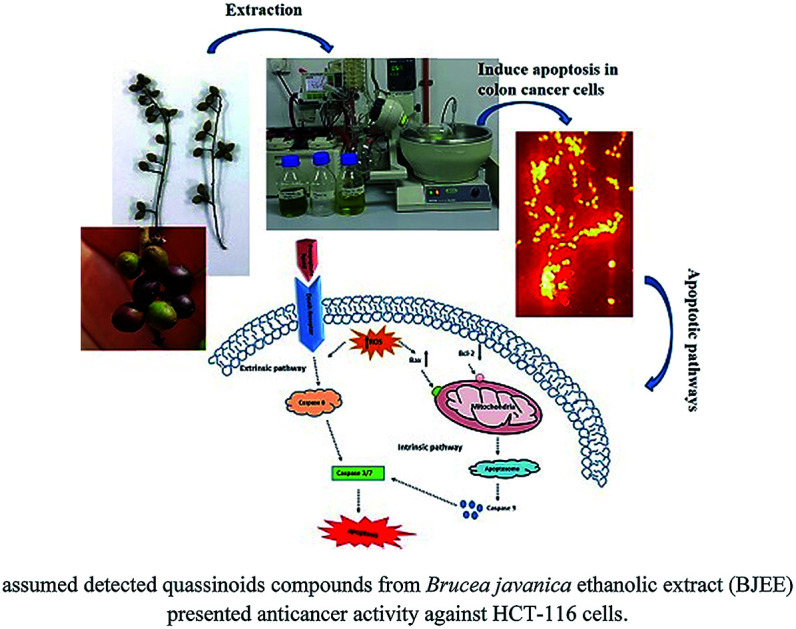

## Introduction

Colorectal cancer (CRC) is a debilitating disease that is known as one of the most common cancers in Asia and its prevalence is growing in a number of Asian countries. However, as yet there are no national or regional guidelines on prevention and screening for early diagnosis of this important disease.^1^ Of the various types of cancer, colorectal cancer is the third most common form in both males and females and the second leading cause of cancer-related deaths worldwide. Many Asian countries have experienced an increase of two to four times in the incidence of colorectal cancer during the past few decades.^2^

Dietary changes, including intake of fresh fruits, vegetables and plants containing high rate of antioxidant are leading methods of CRC prevention,^3^ while obesity may increase the rate of colorectal cancer then physical activities have reverse effects.^4^ Chemotherapy and surgery are the most common available therapeutic treatments, but patient were afflicted with severe side effects, including hair loss, bleeding, immunosuppression and diarrhea, which made the process of treatment more complicated.^5^

Numerous studies have highlighted the potential activates of plants and phytochemicals against diseases including cancer.^6^ Botanicals have been used for the treatment of various human diseases throughout history. Many anticancer drugs are obtained from natural sources. Traditional Chinese Medicine (TCM) is one of the alternative treatment options for modern therapies currently practiced worldwide.^7^ Nature produces some diverse chemical compounds that are often used as anticancer drugs. In spite of their clinical toxicity, they exhibit pharmacological effects and have been used as important traditional remedies for different stages of cancer. In Asian countries, extracts from plants have long been used as an anti-tumor treatment.^8^ In the treatment of cancer, the ideal treatment is one that possesses antitumor properties with minimal toxicity and has a defined mechanism of action. When a natural product that targets specific signalling pathways are identified, researchers can envisage novel therapeutic approaches as well as a better understanding of the pathways involved in disease progression.^6,9^

*Brucea javanica* L. Merr. (*B. Javanica*), known locally in Malaysia as “Lada pahit” is a medicine plant distributed widely through Asia where its habitat includes open areas, secondary forest. Traditionally used in Chinese traditional remedy, bitter fruits of *B. javanica* have been shown to possess anticancer properties. Its fruit extract has been revealed to have anti-proliferative and pro-apoptotic activities on human carcinoma cells.^10^ Recent studies investigated cytotoxicity effects of *B. javanica* on tumor and cancer cells such as lung, bladder and pancreatic cancers.^11^ Its antitumor activities are of research interest, as it is found to have low toxicity but high anti-cancer efficiency^12^ and hence current study was carried out on the ethanolic extract to investigate its anti-proliferative effects on colon cancer cell lines.

## Experimental

### Extraction

The fruits of *B. javanica* were collected from Rimba Ilmu botanical garden, University of Malaya, a Herbarium (KLU) sample deposited with the number KLU.48132. The *B. javanica* fruits were air dried and ground, the plant material (100 g) was extracted by the method described by Kim *et al.* (2016) with some modifications.^13^ First, milled fruits were defatted with hexane and then extraction was done with ethanol by cold maceration method three times and solutions were subsequently filtered, concentrated in a rotary evaporator at 40 °C to produce *B. javanica* ethanol-extract (BJEE).

### Liquid chromatography-mass spectrometry (LC/MS Q-TOF) analysis

Liquid chromatography-mass spectrometry (LC-MS) was used to determine the chemical constituents of BJEE.^13^ An Agilent 6550 ifunnel Q-TOF MS equipped with dual AJS ESI as the ion source was coupled to an Agilent 1200 infinity series HPLC system. Chromatographic separation was carried out using an Agilent Zorbax Eclipse Plus C18 column Rapid Resolution HT (4.6 × 100 mm, 3.5 micron). Running conditions were as follows: solvent composition was consisted of the mixture of two mobile phases A (0.1% formic acid (FA) in water) and B (0.1% FA in 100% acetonitrile). Sample was eluted at a flow rate of 0.5 mL min^−1^ with the ratio of 90% in A at minute 0; 90% in A at 1 minute, 50% in A at 20 minutes, 50% in A at 24 minutes, 90% in A at 25 minutes, and 90% A at 30 minutes with the ratio of 28% in A and 72% in B. The injection volume was 10 μL with column temperature of 40 °C and dual ion modes (dual AJS ESI) were used in MS detection. The detected compounds were recognized from their mass spectra by comparison of the retention times of peaks with interpretation of MS fragmentation patterns from library data.

### Cell lines and culture conditions

Normal human colon epithelial cell CCD-841 CoN (ATCC® CRL-1790™) and human colon cancer cell line HCT-116 (ATCC® CCL-247™) were purchased from the American Type Culture Collection (ATCC, Manassas, VA, USA) and cultured in RPMI-1640 (Sigma, St. Louis, Mo, USA) medium with 10% fetal bovine serum (Biowest, USA), 100 U mL^−1^ penicillin and 100 mg mL^−1^ streptomycin (Gibco, Thermo fisher scientific) in a humidified atmosphere with 5% CO_2_ at 37 °C. For all the assays, untreated medium containing vehicle DMSO (0.1%) was used as a negative control.

### Cell viability assay

The cell viability and cytotoxic effect of BJEE was analyzed by using MTT [3-(4,5-dimethylthiazol-2-yl)-2,5-diphenyltetrazolium bromide] assay as previously described.^14^ In brief, HCT-116 cells (5 × 10^3^ cells per well) were seeded in 96 well-plate and treated with different concentrations of hexane and ethanol extract of *B. javanica* then incubated for 24, 48 and 72 hours. The cells were stained with MTT solution (20 μL per well; 5 mg mL^−1^ in phosphate-buffered saline) for 4 hours, after incubation time the media was removed and DMSO was used to dissolve the formazan crystals. A microplate reader (Tecan Infinite 200 Pro) was used to determine the absorbance at 570 nm. 5-Fluorouracil drug (5-FU, Sigma, St. Louis, Mo, USA) was used as a positive control and DMSO (0.1%) was used as a vehicle control in this study. IC_50_ value was calculated by the following formula;Inhibition (%) = (OD untreated − OD treated)/OD untreated × 100

### Lactate dehydrogenase (LDH) release assay

LDH Cytotoxicity Assay Kit (Promega, USA) was used to detect LDH release from treated colon cancer cells as previously described.^15^ Briefly, HCT-116 cells were seeded at 96-well plates and treated with different concentrations of BJEE for 24 hours. To determine the LDH leakage, Lysis solution was added to 3 wells as positive solution which released the maximum LDH. CytoTox-ONE™ Reagent (100 μL) was added to the wells for 10 minutes at room temperature. The LDH activity was measured by recording fluorescence with an excitation wavelength of 560 nm and an emission wavelength of 590 nm using a Tecan Infinite 200 Pro (Tecan, Männedorf, Switzerland) microplate reader.

### Acridine orange and propidium iodide staining

Dual-fluorescence staining using acridine orange (AO) and propidium iodide (PI) was used to detect early and late apoptosis. It is a nuclear staining method that AO is able to stain both live and dead nucleated cells and generates green fluorescence, while PI only permeate into dead cells with damaged membranes and excite red fluorescence. Briefly, HCT-116 cells were treated with different concentration of BJEE (10, 20 and 40 μg mL^−1^) for 24 hours. The cells were harvested and stained with AO/PI dyes then observed under a UV-fluorescent microscope (Olympus BX51) within 30 minutes.^16^

### Reactive oxygen species (ROS) assay

ROS assay will define the effect of BJEE on reactive oxygen species (ROS) generation in colon cancer cells.^17^ Briefly, HCT-116 cells were cultured on coverslips with complete medium for 6 hours, with the cells exposed to BJEE at IC_50_ concentration and DMSO (negative control) for 6 hours. After incubation time, the treated cells were washed twice with PBS and then stained with dihydroethidium (DHE) (2.5 μg mL^−1^, 1 mL) for 20 minutes at 37 °C. Lastly, cover slips were mounted in a fluorescence microscope slides. Formation of ROS in treated & untreated cells was measured using a fluorescence microscope (Olympus BX51). The level of ROS generation was identified by fluorescence intensity using a commercial kit, Cellular Reactive Oxygen Species Detection Assay Kit (Abcam, Cambridge, UK; ab113851). The HCT-116 cells were treated with different concentration of BJEE (2.5, 5, 10 and 20 μg mL^−1^) or DMSO (negative control) in a 96-well plate for 6 hours according to the manufacturer's protocol. The fluorescence intensity was determined by fluorescent microplate reader (Tecan Infinite M 200 PRO, Männedorf, Switzerland) at wavelength of 500 nm excitation and 580 nm emission.

### Caspase activity assay

Activation of caspase was measured by caspase-Glo assay (Promega, USA), as per the manufacturer's protocol. Briefly, HCT-116 cells were seeded in a white walled 96-well plate and cells were allowed to grow to reach 80% confluency before treatment with different concentration (2.5, 5, 10 and 20 μg mL^−1^) of BJEE and incubated for 24 hours. After 24 hours, cells were mixed with caspase reagents (caspase 3/7, caspase 8, caspase 9) then sealed plate was incubated for 1 hour at room temperature. The luminescence of each sample was measured in a plate-reading luminometer (GloMax microplate luminescence reader, Promega Company, USA).^18^

### Gene expression analysis by real time RT-PCR

The HCT-116 cells were seeded in a T25 cm^2^ flasks, and allowed to reach 80% confluency. Cells were treated with BJEE at IC_50_ concentration for 24 h. Commercial RN easy mini kit (Qiagen, Germany; 74106) was used to extract RNA from treated and untreated cells. The quality of RNA was distinguished by NanoDrop 2000c with the 260/280 ratios (>1.8), and by gel electrophoresis to visualize the integrity of 18S and 28S bands. The total RNA was reversed to cDNA by using high-capacity RNA-to-cDNA kit, according to the manufacturer's protocol.

The cDNA conversion was carried out using the TaqMan® Gene Expression Master Mix by following the manufacturer's protocol. The expression of two endogenous controls primers GAPDH (Hs03929097_g1) and ACTB (Hs99999903_m1) were used in this study. The specific primers including caspase 8 (Casp8, Hs01018151_m1), caspase 9 (Casp9, Hs00609647_m1), caspase 3 (Casp3, Hs00234387_m1), BCL2 (BCL2, Hs00608023_m1) and BCL2 associated X (BAX, Hs00180269_m1) were bought from TaqMan® (MGB probes, FAM™ dye-labeled). All samples were loaded into three wells for biological triplicate experiments. The ΔΔCt method was used to calculate the relative changes in gene expression specified from RT-PCR.^19^

### Statistical analysis

All the experimental results were presented as means ± SD, and all measurements and analysis were carried out in triplicate for *in vitro* studies. Excel 2010 and SPSS (version 18.0) statistical software, one-way analysis of variance (ANOVA) with Tukey's multiple comparisons and the Student's *t*-test were used for the statistical and graphical evaluation. All *p*-values < 0.05 was considered statistically significant.

## Results and discussion

### Low concentration of BJEE inhibited growth of HCT-116 colorectal cancer cells

The cytotoxic effects of hexane and ethanol extract of *B. javanica* on HCT-116 cancer cells and CCD-841 CoN normal colon cells were tested by using the MTT assay. Ethanol extract showed a significant and higher cytotoxicity on HCT-116 cancer cells (Table 1) compared to hexane extract (data not shown). The MTT results from hexane extract were not presented in the results table. The IC_50_ values of the *B. javanica* ethanolic extract (BJEE) were determined at three different treatment time points (24, 48 & 72 hours) from three independent MTT experiments. The results as presented in Table 1 elicited the strongest cytotoxicity and inhibitory effect of BJEE on treated cells (HCT-116). In contrast, even high concentration of BJEE (400 μg mL^−1^) did not show significant effect on proliferation of normal colon cells (CCD-841 CoN) after treatment.

**Table 1:** Inhibitory effects of BJEE on the proliferation of human normal and cancer colon cells^a^

IC_50_ (μg mL^−1^)				
Cell line	Classification	24 hours	48 hours	72 hours
HCT-116	Colon cancer cells	8.9 ± 1.32*	5.2 ± 0.98*	2.7 ± 1.06*
CCD-841 CoN	Colon normal cells	>900	>400	>400

a Cells were treated with different concentrations of BJEE for 24, 48, and 72 hours. The IC_50_ values represent the concentration of the BJEE that could inhibits the 50% cells growth. *p ≤ 0.05 compared to the control at the corresponding time.

Findings from this study were comparable with previous studies, which showed cytotoxic and apoptotic effects of *B. javanica* extracts against the T24 bladder cancer cell line,^11^ H1975 cells (human lung cancer cells),^13^ PANC-1, pancreatic adenocarcinoma cell lines (SW1990 and CAPAN-1),^20^ MCF-7 (mammary adenocarcinoma cell line), LNCaP (the prostate carcinoma cells) and the human epidermoid carcinoma cell line (A431).^21^ Furthermore, some studies have suggested no inhibitory effect of *B. javanica* extract on normal cells *i.e.* Human Mammary Epithelial Cell (HMEC) and *Cercopithecus aethiops* monkey kidney normal cell line (CV-1).^21^ Our finding from this study showed significant cytotoxic effect of ethanol extract of *B. javanica* (BJEE) against colorectal cancer cells (HCT-116). In contrast, there was no cytotoxicity on normal colon cells (CCD-841 CoN) even at high concentration of BJEE.

### BJEE induced LDH release

**Fig. 1:**
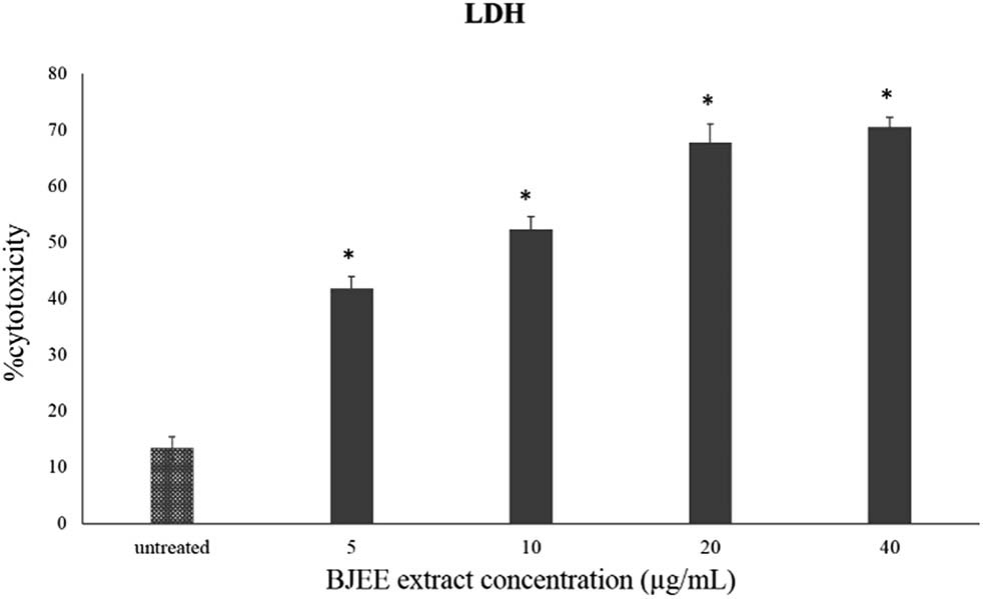
LDH assay showed the cytotoxicity percentage of BJEE towards HCT-116 cells. The data represent the means ± SD (*p ≤ 0.05) of three independent experiments.

In this study, we measured the level of LDH leakage from HCT-116 cells into medium to assess the cytotoxicity percentage of ethanol extract after 24 hours of treatment. As depicted in Fig. 1, cytotoxicity of BJEE is dose-dependent and the release of LDH into medium was at 5 to 40 μg mL^−1^ concentration. The results of MTT and LDH assays together confirm the obvious cytotoxic & anti-proliferative effect of BJEE towards HCT-116 cells.

LDH release is a marker that is released in damaged cell membranes including necrosis and apoptosis.^22^ Hence, measurement of the LDH leakage from cells into the medium is an indicator to identify the cytotoxicity of anti-cancer drugs.^23^ The results of MTT and LDH assays together confirm the obvious cytotoxic & anti-proliferative effect of BJEE towards HCT-116 cells.

### Phytochemical screening of BJEE

**Fig. 2:**
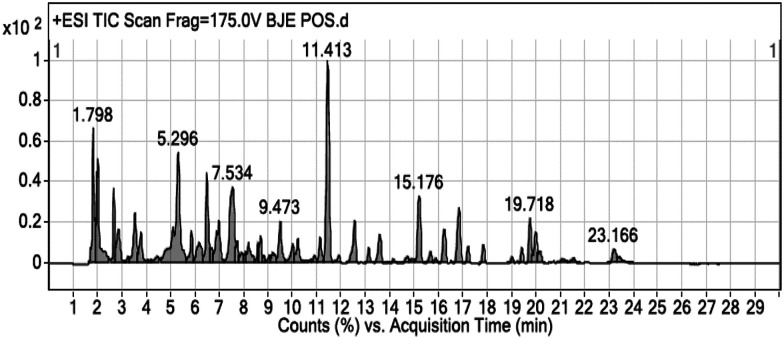
Chromatographic fingerprint analysis of the BJEE. Thirteen major compounds in BJEE were identified by LC/MS Q-TOF. Abbreviations: BJEE, *Brucea javanica* ethanol extract; LC/MS, liquid chromatography/mass spectrometry.

The results from cell viability assay showed ethanol extract of *B. javanica* with potent anticancer activities on colon cancer cells (HCT-116). The BJEE was selected therefore for phytochemical screening to know the phytoconstituents present in the extract. As shown in Fig. 2, the chemical composition of BJEE was characterized using Liquid Chromatography-Mass Spectrometry Quad Time of Flight (LC/MS Q-TOF) analysis. Thirteen major components were identified from the chromatogram that is inclusive of 1: 4-fluoro-l-threonine, 2: trolamine, 3: vidarabine, 4: oxyquinoline, 5: bruceine D, 6: 2′′′′,2′′′′′,2′′′′′′-trihydroxy-5′′,5′′′′,5′′′′′-tribenzyldiuvaretin, 7: castillene B, 8: quassimarin, 9: isobrucein A, 10: alpha-heptasaccharide, 11: C16 sphinganine, 12: phytosphingosine, and 13: enigmol (Table 2).

**Table 2:** LC-MS-QTOF analysis determined nine detected compounds in BJEE

No.	Name of compounds	RT (min)	Mass	Formula
1	4-Fluoro-l-threonine	1.88	137.0492	C4H8FNO3
2	Trolamine	2.059	149.1059	C6H15NO3
3	Vidarabine	2.644	267.0971	C10H13N5 O4
4	Oxyquinoline	5.29	145.0532	C9H7NO
5	Bruceine D	6.542	410.1579	C20H26O9
6	2′′′′,2′′′′′,2′′′′′′-Trihydroxy-5′′,5′′′′,5′′′′′-tribenzyldiuvaretin	7.05	802.3153	C51H46O9
7	Castillene B	9.499	310.1207	C19H18O4
8	Quassimarin	13.56	522.2109	C26H34O11
9	Isobrucein A	15.17	522.2111	C26H34O11
10	Alpha-heptasaccharide	16.8	1202.4437	C46H78N2O34
11	C16 sphinganine	19.71	273.2681	C16H35NO2
12	Phytosphingosine	19.94	317.294	C18H39NO3
13	Enigmol	23.14	301.2979	C18H39NO2

During the years of studies on *Brucea javanica* numerous compounds with their concentration have been detected and isolated from this plant, consisting of quassinoids, alkaloids, triterpenes, steroids, coumarins, anthraquinones, flavonoids and other metabolites.^24^ In this study thirteen components with high peaks were detected from ethanolic extract of *B. javanica* which we assumed six of them presented anticancer activities according to the earlier studies. Previous investigations on *Brucea javanica* revealed the main compound with anti-tumor activity belongs to quassinoids which showed anti-cancer activity against various type of cancer cells such as: HCT-8, HepG2, BGC-823, A549, and SKVO3 and due to this earlier study, inhibitory effect of quassinoid compounds was observed against one type of colon cancer cell line (HCT-8).^25^ Results from LCMS analysis in this study indicated presence of three major compounds derived from quassinoid (bruceine D, isobrucein A, and quassimarin) with different percentage of (5%, 15%, 30% respectively). The other three major compounds that were detected by LCMS derived from sphingolipids include: C16 sphinganine 13%, phytosphingosine 11%, and enigmol 9% with capability of apoptosis induction due to the previous studies.^26,27^

Previous studies showed bruceine D is the main component from *Brucea javanica*. The pharmacological studies on this quassinoid derivative compound revealed anti-proliferative and apoptogenetic action in different type of cancers including pancreatic,^28^ and hepatocellular carcinoma.^29^ Brucein-D showed anti-cancer activity as well against human pancreatic adenocarcinoma cells by mediating the p38-mitogen-activated protein kinase and inhibition of NF-κB activity in cancer cells.^30^ Isobrucein from another quassinoid derivative showed anti-inflammatory and antihyperalgesic activities in earlier studies.^31^ The compound isobrucein-A also possesses growth inhibitory activities against the P-388 lymphocytic leukemia *in vivo*, and the Eagle's carcinoma of the nasopharynx (KB) system *in vitro*.^32,33^ Quassimorin known as one of earliest detected compound of quassinoid derivatives suppressed the growth of a panel of human tumor cell lines (KB, A-549, HCT-8, CAKI-1, MCF-7, and SK-MEL-2)^34^ and has been identified as an antileukemic compound.^35^

The sphingoid bases are cytotoxic for many cancer cell lines and contributed to suppression of intestinal tumorigenesis by ingested sphingolipids.^36^ Phytosphingosine is a highly bioactive compound derived from sphingosine which induced apoptosis, *via* caspase 8 activation and Bax translocation.^37^ C16 sphinganine another component from sphingolipids showed anticancer activity in HCT-116 cells.^38^ Enigmol from sphingolipids was showed a potential anticancer activity against colon cancer cell line (HT29).^39^ It also suppressed tumor growth in the prostate cancer cell lines (DU145 and PC-3) and decreased the number of intestinal tumors in Min mice.^40^

### Microscopy and AO/PI double staining

**Fig. 3:**
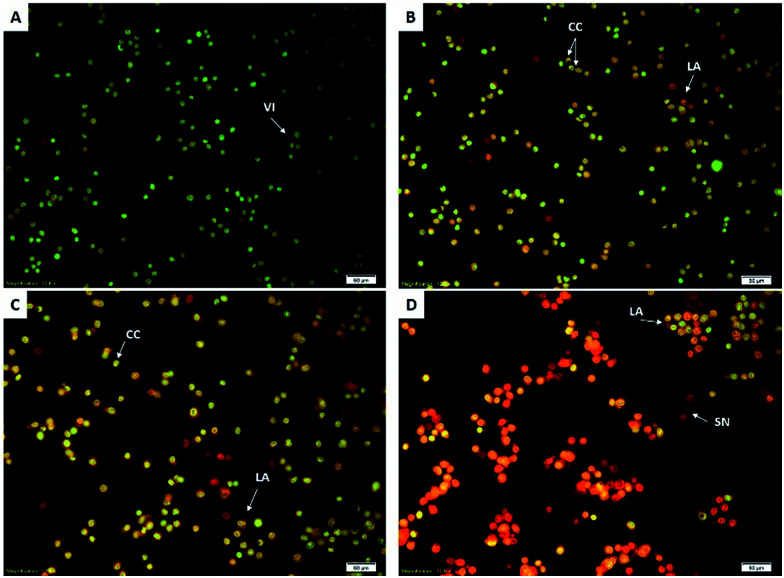
Fluorescent micrograph of acridine orange and propidium iodide double-stained human HCT-116 cells. Cells were treated with different concentrations (10, 20, 40 μg mL^−1^) of BJEE for 24 hours. (A) Untreated HCT-116 cells after 24 hours demonstrated normal structure without prominent apoptosis. (B) Treated cells at concentration of 10 μg mL^−1^ BJEE showed early apoptosis features, including chromatin condensation. (C) Early and late apoptosis were observed at concentration of 20 μg mL^−1^ and (D) late apoptosis and bright red colored secondary necrosis was obvious after 24 hours of BJEE treatment at 40 μg mL^−1^ (magnification: 20×). Abbreviation: VI: viable cells; CC: chromatin condensation; LA: late apoptosis; SN: secondary necrosis; BJEE, *Brucea javanica* ethanol extract.

Acridine orange and propidium iodide staining is a method to study the morphological changes of the cells. HCT-116 cells treated with different concentrations of BJEE (10, 20, 40 μg mL^−1^), were observed under a fluorescent microscope to analyse the viable cells, early apoptosis, and late apoptosis (Fig. 3). Untreated HCT-116 cells after 24 hours showed viable cells with green colour and intact nuclei. In contrast, early apoptosis was observed in treated cells with BJEE at 10 and 20 μg mL^−1^. The late stage of apoptosis was exhibited at 20 and 40 μg mL^−1^ concentration of BJEE (Fig. 3). The results indicated that BJEE has a dose-dependent apoptogenic effect.

Acridine orange and propidium iodide staining is a method to study the morphological changes of the cells. AO stain with green fluorescence color penetrated from plasma membrane of viable cells or early apoptotic cells with fragmented DNA. AO was obvious with bright-green nuclei and chromatin condensation at early stage of apoptosis. Further, PI stain displayed dead cells, while orange nuclei are present at the late stage of apoptosis due to the binding of AO to denatured DNA.^16^ Specific changes in morphological characterizations of cells indicated apoptosis occurrence. These changes include cell shrinkage, nuclear or cytoplasmic fragmentations, chromatin condensation and formation of dense bodies that are phagocytized by neighboring cells.^41^ The early apoptosis and late apoptosis occurrence could be observed by fluorescent microscopy analysis and AO/PI double staining.^42,43^ In this study, treated HCT-116 cells with BJEE illustrated some signs of apoptosis, namely, cytoplasmic shrinkage, membrane blebbing, and DNA fragmentation.

### BJEE elevated reactive oxygen species (ROS) production level

**Fig. 4:**
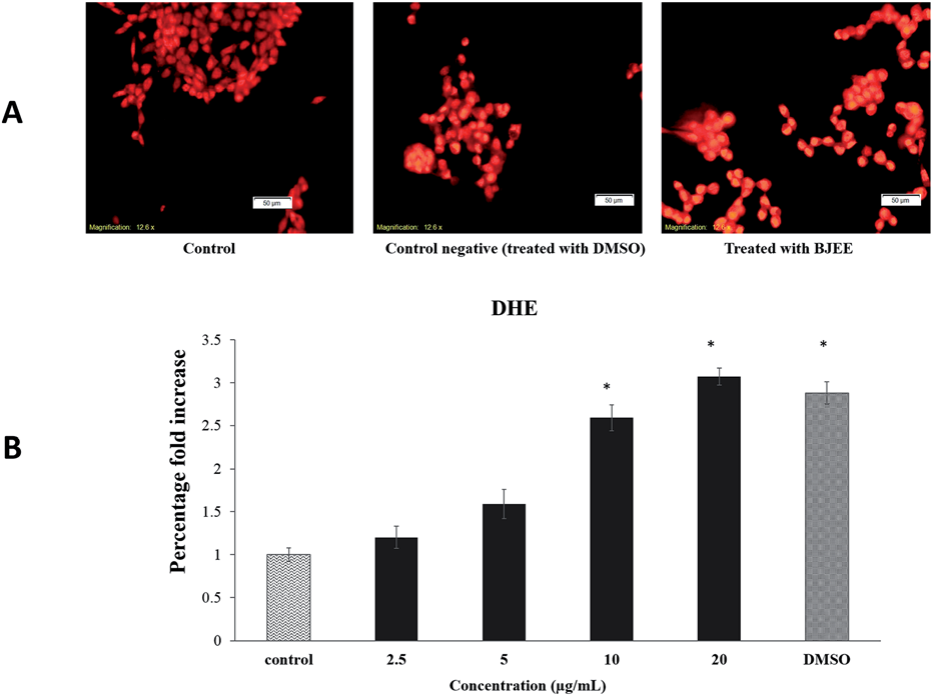
Effect of BJEE on ROS generation in HCT-116 cells. (A) HCT-116 cells were treated with BJEE at IC_50_ concentration (9 μg mL^−1^) and DMSO as negative control for 6 hours then stained with DHE dye (red) (magnification: 20×). (B) A bar chart represents significant effect of BJEE elevated ROS production at concentration of 10 to 20 μg mL^−1^ after 6 hours. The data represents the means ± SD of three independent experiments. *p ≤ 0.05 compared with the untreated group. Abbreviations: BJEE, *Brucea javanica* ethanol extract; DMSO, Dimethyl sulfoxide.

In our experiment, we have tested the influence of BJEE on ROS production level. As shown in Fig. 4A and B, the results of ROS assay reveal that BJEE affect the level of intracellular ROS by a dose-dependent manner. Fig. 4A, depicted ROS production and is concentrated in HCT-116 cells in comparison to untreated one. According to the results ROS level was significantly increased in treated cells with BJEE at concentration of 10 and 20 μg mL^−1^ (Fig. 4B). Our results from this study demonstrated increase in the level of intracellular ROS occurred in HCT-116 cells after treatment with BJEE.

Reactive oxygen species (ROS) generation play an important role in the cell signalling and activation of mitochondria-initiated events leading to apoptosis.^44^ Over production of ROS can be a cause of oxidative damage to lipids, proteins and DNA of cells which can lead to tumor genesis or cell death.^45^ Previous studies showed the critical role of mitochondria in the regulation of cell death and survival.^46^ The excessive production of ROS can cause damage to mitochondrial membrane phospholipids, leading to open transition pores and decrease in the mitochondrial membrane permeability. Damage to the potential efficiency of the mitochondrial membrane is caused at early stage of apoptosis.^47^ The results obtained from Fig. 4 showed the significant elevation of ROS level in treated cells which may cause of mitochondrial dysfunction in cells and apoptosis occurrence. In addition, our results from this assay are in the line with previous research study, which indicated the apoptotic effect of *Brucea javanica* seed oil on acute myeloid leukemia cell line (HL-60) through a mitochondria-mediated pathway due to ROS production.^48^

### Caspase activation induced by BJEE

**Fig. 5:**
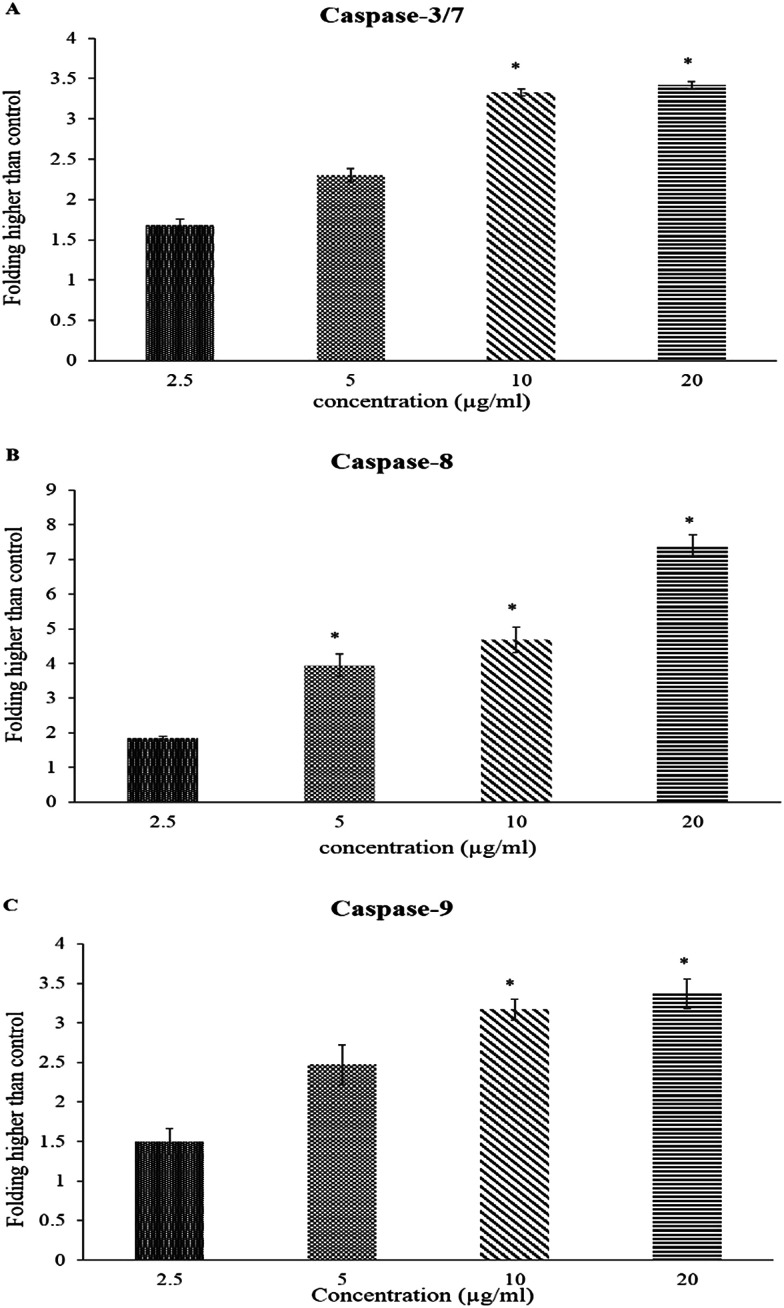
Relative expression of caspase-3/7 (A), caspase-8 (B) and caspase-9 (C) in the HCT-116 cells treated with various concentrations of BJEE. Triplicates of each treatment group were used in each independent experiment. The data represent the means ± SD of three independent experiments; statistical significance is expressed as, *p ≤ 0.05. (A) Caspase-3/7, (B) caspase-8 & (C) caspase-9. Abbreviation: BJEE, *Brucea javanica* ethanol extract.

The caspase-Glo-8 and -9 assays were used to identify the pathway activated upon treatment of the tumorigenic cell line (HCT-116) with BJEE. As shown in Fig. 5, the achieved results illustrated an increase of the enzyme activities in a dose-dependent manner. HCT-116 cells were treated with different concentrations of BJEE for 24 hours. The results in Fig. 5 showed significant elevation in the activity of caspase-3/7 and 9 at the 10 and 20 μg mL^−1^ concentrations, while caspase-8 showed significant elevation with the highest folding in various concentrations (5, 10 and 20 μg mL^−1^) compared to caspase 3/7 and 9. The results showed that the extract was able to induce apoptosis by caspase-3/7 (Fig. 5A). Caspase-8 (Fig. 5B) and caspase-9 (Fig. 5C) showed significant activities in treated cells in comparison to untreated one.

The caspase cascade signaling system is an important factor in the induction of apoptosis, which is accurately controlled by different type of proapoptotic and antiapoptotic molecules.^49^ The caspase-Glo-3/7 assay targets both caspase-3 and caspase-7, as they identify the same peptide for cleavage and the substrate in the kit is a peptide that becomes luminescent on cleavage.^50^ Previous studies showed that caspase-9 is the primary activation step that cascades to the mitochondrial pathway which eventually results in apoptosis^51^ and caspase-8 is a member of executioner caspases associated with tumor necrosis factor (TNF) and death receptors-mediated apoptotic signaling cascade.^52^ According to the caspase study results, the apoptosis-inducing potential of BJEE through the mitochondrial pathway was considered by significant activation of caspase-9. Further, excessive elevation of ROS is associated with activation of downstream production such as caspase-9, which is potentially recommended mitochondrial pathway for induction of apoptosis. In addition, significant and highest elevation of caspase-8 after treatment with BJEE indicated possible contribution of extrinsic pathway with apoptosis-induction. Involvement of more than a single apoptotic pathway in the induction of apoptosis by anticancer compounds is well-established in the literature, especially by natural compounds derived *via* plant extract, such as curcumin.^53,54^

### BJEE elevated gene expression of Bax, Casp3, 9 & 8

**Fig. 6:**
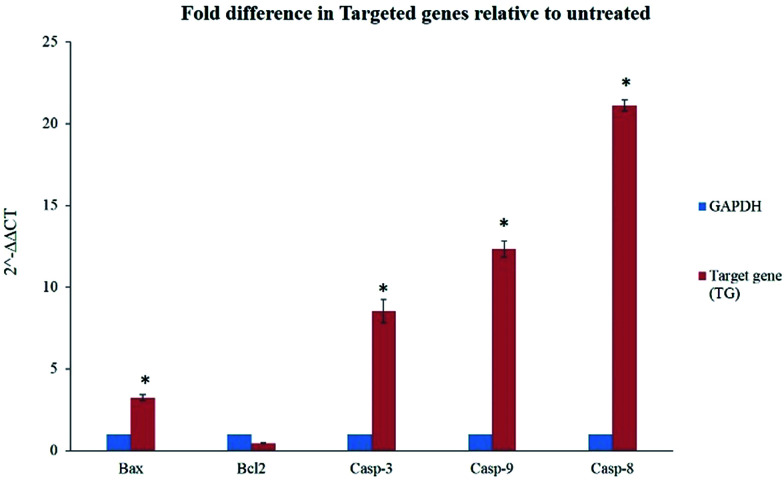
Quantitative analysis of gene expression in HCT-116 treated cells with BJEE after 24 hours showed a significant fold differences in Bax, Casp-3, 9 & 8 genes relative to untreated cells. Meanwhile, gene expression of Bcl-2 did not elicit significant changes. Statistical significance is expressed as, *p ≤ 0.05. Abbreviation: BJEE, *Brucea javanica* ethanol extract.

The gene expression results in this study showed significant elevation of Bax gene expression, also supported by our findings from caspase and ROS assay. However, Bcl-2 gene was not significantly downregulated but its expression was inhibited in treated cells with BJEE (Fig. 6). This study had showed 21 folds of Casp-8 in treated cells that endorses its role in mediating extrinsic apoptosis in treated cells. In addition, Casp-9 & Caps-3 were overexpressed 12 & 8 folds respectively in treated cells (HCT-116) with BJEE, which may demonstrate interference of mitochondria pathway in apoptosis (Fig. 6).

The earlier studies exhibited the proapoptotic protein (Bax) from BCL2 family induces caspase activation and ROS production in cells.^55^ In case of irreparable DNA damage, the overexpression of pro-apoptotic gene like Bax would have occurred in cells, which triggered downregulation of anti-apoptotic gene like Bcl-2.^19^ Caspase pathways and mitochondria dysfunction were executed when the expression of Bcl-2 gene was inhibited by up-regulation of Bax.^56^ Inhibition of anti-apoptotic member (Bcl-2) is the trigger of intrinsic apoptosis pathway.^57^ Caspase-9 overexpression subsequently activates caspase-3 which leads to cell apoptosis *via* intrinsic pathway.^58,59^ Recent studies on *Brucea javanica* oil showed apoptosis was induced in bladder cancer cells *via* activation and overexpression of caspase-3 and caspase-9.^11^ Moreover, caspase-8 gene (Casp8) also plays a key role in cell apoptosis.^60,61^ Upregulation of Casp-8 gene followed by caspase-3 activation induced extrinsic apoptosis pathways *via* Fas-ligand activation.^62,63^ In addition, previous studies from some of *Brucea javanica* extracts such as aqueous and oil extracts, indicated caspase-8, and mitochondrial-pathways mediated into apoptosis of cancer cells.^11,13,48^

According to the previous findings and our obtained results from RT-PCR demonstrated in Fig. 6, we may have considered interferences of mitochondria-intrinsic and extrinsic pathways in apoptosis induction on treated HCT-116 cells with BJEE. Involvement of more than a single apoptotic pathway in the induction of apoptosis by anticancer agents is well-established in the literature, especially by natural compounds derived *via* plant extract, such as curcumin.^53,54^

## Conclusion

In conclusion, the plant extract of *Brucea javanica* has been found to have the potential anti-proliferation and apoptotic activity in HCT-116 colorectal carcinoma cells. The apoptosis induced in HCT-116 cells is *via* mitochondria and extrinsic pathway through activation of caspase enzyme and upregulation of Bax, Casp-3 and Casp-9 genes. The active role of mitochondria in the induction of apoptosis was confirmed by excessive production of ROS. Caspase activity and gene expression analysis from this study also revealed elevation of Casp-8 gene, with this expression contributing to extrinsic pathway. Moreover, six major compounds are detected from BJEE, which three of them derived from quassinoids such as bruceine D, isobrucein A, and quassimarin with different percentage of (5%, 15%, 30% respectively), and the other three major compounds derived from sphingolipids include: C16 sphinganine 13%, phytosphingosine 11%, and enigmol 9%. These six detected compounds may probably be responsible to induce apoptosis in HCT-116 cells. Finally, our findings illustrated promising anticancer activity of BJEE against HCT-116 colon cancer cells. However, further *in vitro* and *in vivo* studies on the probable active compounds of this plant responsible for the above activities and the mechanistic studies based on them are still required to be investigated.

## Author contributions

E. B. Executed *in vitro* experiments, analysis of the data; and writing of the manuscript. F. H. Data analysis and helping to rewrite some parts of the paper. S. N., principal investigator, contributed to the design of work, critical review of study protocol and editing of manuscript and review of the paper before submitting to journal. N. A. S. Co-investigator of the project, intellectual contribution, conceived the idea, and editing of manuscript.

## Conflicts of interest

The authors revealed there are no conflict of interest. The funding sponsors had no role in the design of the study, data collection and analysis, in the writing of the manuscript, or in the decision to publish the results.

## Supplementary Material
